# Supplementary material for: Identification of Genes Associated with Gut Microbiota Metabolites in Type 2 Diabetes Mellitus Complicated by Acute Cerebral Infarction Based on Bioinformatics and Mendelian Randomization
Source: Biomedicines. 2026 Oct 17;14(10):2371. doi: 10.3390/biomedicines14102371 (PMC13702449; doi:10.3390/biomedicines14102371)
Supplement: Supplementary file 1 [file biomedicines-14-02371-s001.zip › Figure S4/genus.Eisenbergiella.id.11304 ACI.pdf]

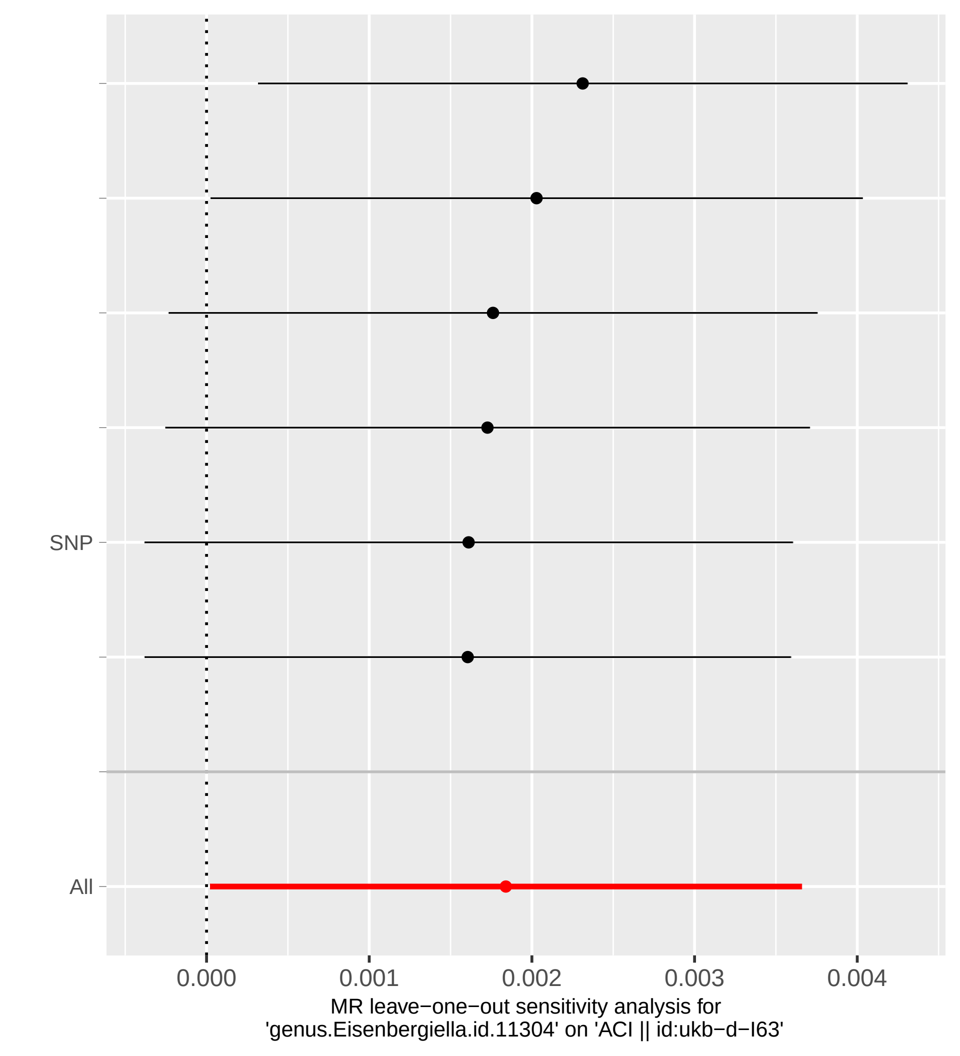
SNP
All
0.000
0.001
0.002
0.003
0.004
MR leave−one−out sensitivity analysis for
'genus.Eisenbergiella.id.11304' on 'ACI || id:ukb−d−I63'
